# Mating type‐dependent partner sensing as mediated by VEL1 in *T* *richoderma reesei*

**DOI:** 10.1111/mmi.12993

**Published:** 2015-04-16

**Authors:** Hoda Bazafkan, Christoph Dattenböck, Stefan Böhmdorfer, Doris Tisch, Eva Stappler, Monika Schmoll

**Affiliations:** ^1^ Department Health and EnvironmentAIT Austrian Institute of Technology GmbHBioresourcesKonrad‐Lorenz Strasse 24Tulln3430Austria; ^2^ University of Natural Resources and Life Sciences ViennaDepartment of ChemistryDivision of Chemistry of Renewable ResourcesKonrad‐Lorenz‐Straße 24Tulln3430Austria; ^3^ Research Area Molecular BiotechnologyVienna University of TechnologyGumpendorfer Straße 1aWien1060Austria

## Abstract

Sexual development in the filamentous model ascomycete *T* *richoderma reesei* (syn. *H* *ypocrea jecorina*) was described only a few years ago. In this study, we show a novel role for VELVET in fungi, which links light response, development and secondary metabolism. *V* *el1* is required for mating in darkness, normal growth and conidiation. In light, *vel1* was dispensable for male fertility but essential for female fertility in both mating types. VEL1 impacted regulation of the pheromone system (*hpr1*, *hpr2*, *hpp1*, *ppg1*) in a mating type‐dependent manner and depending on the mating partner of a given strain. These partner effects only occurred for *hpp1* and *hpr2*, the pheromone precursor and receptor genes associated with the MAT1‐2 mating type and for the mating type gene *mat1‐2‐1*. Analysis of secondary metabolite patterns secreted by wild type and mutants under asexual and sexual conditions revealed that even in the wild type, the patterns change upon encounter of a mating partner, with again distinct differences for wild type and *vel1* mutants. Hence, *T* *. reesei* applies a language of pheromones and secondary metabolites to communicate with mating partners and that this communication is at least in part mediated by VEL1.

## Introduction

Sexual reproduction has developed and spread in evolution as a mechanism to survive challenging environmental circumstances and to improve competitiveness by increased phenotypic variability, despite the higher energetic costs and the risk to lose a successful phenotype due to recombination. Heterothallic ascomycetes, which need a mating partner of opposite mating type for sexual reproduction, can assume the male or female role in a cross and are hence considered hermaphroditic (Debuchy *et al*., 2010; Nieuwenhuis and Aanen, 2012). However, the distinction between male or female and sex determination by mating type are not equally clear in all fungi and consequently, it is a matter of debate whether mating type and sex can indeed be generally separated in fungi (Heitman *et al*., 2013). Assuming the female role thereby means a considerable investment in terms of resources to form female reproduction structures, which may or may not be fertilized by compatible males. Nevertheless, recombination during meiosis can also serve as the only escape strategy to survive decaying environmental conditions as a species (Aanen and Hoekstra, 2007). In fungi, it is debated if ‘real’ asexual species exist or if their sexual stage has just not been discovered yet, hence emphasizing the importance of sexual development (Taylor *et al*., 1999).

The filamentous ascomycete *Trichoderma reesei* (syn. *Hypocrea jecorina*) represents a workhorse for numerous biotechnological applications, which are nowadays mainly targeted at improvement of production of second‐generation biofuels (Schmoll *et al*., 2014). Decades of strain improvement with *T. reesei* resulted in detailed knowledge of nutrient requirements, regulation mechanisms and regulators of enzyme production (Kubicek *et al*., 2009; Schmoll *et al*., 2014). In recent years, also signal transduction pathways, especially those transmitting light signals, were investigated for their role in enzyme production and development in *T. reesei* (Schmoll *et al*., 2010a; 2014).

In *T. reesei*, the discovery of sexual development was reported a few years ago (for an overview, see Schmoll, 2013). The possibility to cross strains with different characteristics added a valuable tool for investigation of *T. reesei*. However, a QM6a derivative, which contained the other mating type, was not able to form fruiting bodies with QM6a, although the same experiment enabled fruiting body formation in the wild‐type strain CBS999.97. Hence, QM6a was proposed to be female sterile (Seidl *et al*., 2009). This finding that QM6a, the parent strain of all strains used in research and industry, is female sterile hampers applications of sexual development in strain improvement and is subject to continued research. So far, no conditions have been reported that would induce formation of protoperithecia with *T. reesei*. Therefore, combinations of female/male fertile and female/male sterile strains have to be used to test for fertility.

For accomplishment of sexual development, the mycelium is required to continuously function as an information network. Availability of nutrients, light or darkness and sensing of compatible partners are crucial for initiation of the sexual stage and hence represent important information that has to be transmitted and interpreted in the cell (Debuchy *et al*., 2010). One of the most crucial signaling systems for communication between potential mating partners, the pheromone system, shows an interesting deviation from other fungi in *T. reesei*. Besides normal orthologs of pheromone receptors (HPR1 and HPR2) and an alpha type peptide pheromone precursor (PPG1), an h‐type peptide pheromone precursor (HPP1) with characteristics of a‐ and alpha type assumes a‐type function (Schmoll *et al*., 2010b). For successful mating, a pair of receptor and cognate pheromone precursor is required. Consequently, either *ppg1* and *hpr2* or *hpp1* and *hpr1* are required for successful mating. Thereby, if one of the pheromones is missing from the genome, the other can take over in both mating types, as their transcription is not strictly mating type dependent (Seibel *et al*., 2012b). However, as in other fungi, lack of the mating type‐associated pheromone receptor (HPR1 in MAT1‐1 or HPR2 in MAT1‐2) leads to female sterility, while pheromone precursors are essential for male fertility in *T. reesei* (Kim and Borkovich, 2004; Seibel *et al*., 2012b). Hence, in contrast to the pheromone precursors, the effect of availability of pheromone receptors is mating type dependent. Transcript levels of *hpp1*, *ppg1* and *hpr1* increase under conditions of sexual development in the wild‐type strain CBS999.97, while for *hpr2*, this is only the case in MAT1‐2 upon fruiting body formation (Seibel *et al*., 2012b).

Sexual development in *T. reesei* is most efficient in daylight (light–dark cycles), but also occurs in darkness with some delay (Seidl *et al*., 2009; Chen *et al*., 2012; Seibel *et al*., 2012a). While the photoreceptors of *T. reesei*, BLR1, BLR2 and ENV1, influence many light responses (Castellanos *et al*., 2010), only ENV1 has an important influence on fruiting body formation. Two mutants of opposite mating types, both lacking *env1*, are not able to undergo sexual development in light, but do not show this defect in darkness. At the transcriptional level, these mutants show extreme overexpression of the pheromone precursor genes in light, especially of *hpp1*. Hence, their defect in light is assumed to be caused by misregulation of the pheromone system and subsequent loss of sexual identity (Seibel *et al*., 2012a).

Since the discovery of VELVET (VeA) in *Aspergillus nidulans*, its homologs were studied extensively and found to be crucial regulators of light‐dependent development in diverse fungi (Bayram and Braus, 2012). VeA is further known to be essential for normal secondary metabolism, particularly with respect to biosynthesis of sterigmatocystin in *A. nidulans* but also for diverse other metabolites in various fungi. This is due to regulation of downstream transcription factors (summarized in Calvo, 2008). The coordination of the light signal with development and secondary metabolism is accomplished by an intricate mechanism (Bayram *et al*., 2008a; Sarikaya‐Bayram *et al*., 2014). Thereby, VeA interacts with the phytochrome FphA in dependence of the presence of the chromophore in FphA, and this interaction is restricted to the nucleus. FphA in turn interacts with the photoreceptor complex (LreA‐LreB) via LreB (Purschwitz *et al*., 2008).

Interestingly, the function of VeA homologs in regulation of asexual development can be negative (Mooney and Yager, 1990; Bayram *et al*., 2008b; Jiang *et al*., 2011; Schumacher *et al*., 2012; Lopez‐Berges *et al*., 2013) or positive (Calvo *et al*., 2004; Hoff *et al*., 2010; Kopke *et al*., 2013; Kim *et al*., 2014). With respect to secondary metabolism, mostly positive regulation by VeA homologs is reported in the studies mentioned above, although in some cases, a negative function on individual metabolites was found (Wiemann *et al*., 2010; Schumacher *et al*., 2013).

In *Trichoderma* spp., the VeA ortholog VEL1 of *Trichoderma virens* was studied, which was found to act positively on secondary metabolism, biocontrol efficiency, morphogenesis and asexual development (Mukherjee and Kenerley, 2010). Recently, a positive effect on development was also observed in a female sterile strain of *T. reesei*, and VEL1 was found to be involved in regulation of cellulase gene expression (Karimi Aghcheh *et al*., 2014).

In this study, we aimed to investigate the role of VEL1 in light‐dependent development in *T. reesei* in detail. We show that VEL1 is essential for sexual development in darkness and required for female fertility in light. Investigation of the pheromone system as influenced by VEL1 suggested a role in communication between potential mating partners. Indeed, we found that VEL1 is involved in regulation of the response to another fungus encountered in terms of signal reception (regulation of pheromone receptor transcripts), signal transmission (pheromone precursors) and chemical communication.

## Results

### VEL1 is required for asexual development

The velvet family is represented in *T. reesei* with VEL1 (TR_122284), VEL2 (TR_40551) and VEL3 (TR_102737), which largely share the characteristics of their homologs in *Aspergilli*, but no VosA homolog is present in the genome (for details, see Supporting Information Appendix S1). For functional analysis, we deleted *vel1* in the wild‐type strain QM6a. The resulting strain did not sporulate on various carbon sources and under different light and stress conditions. VEL1 was found to further impact growth and cellulase expression (Supporting Information Appendix S2). Hence, the phenotype of deletion of *vel1* in the high cellulase mutant strain QM9414 as reported earlier (Karimi Aghcheh *et al*., 2014) was largely confirmed for its parental wild‐type strain QM6a.

### VEL1 is essential for sexual development in darkness

In order to investigate the function of *T. reesei* VEL1 in sexual development in a female fertile strain background, we constructed strains (named FF1 and FF2) derived from female sterile QM6a of both mating types able to undergo mating with a female sterile partner by crossing with the female fertile wild‐type CBS999.97 and repeated backcrossing with QM6a as described earlier (Schuster *et al*., 2012). In agreement with earlier reports (Schuster *et al*., 2012), the difference in sporulation between growth in light and darkness was larger in these strains compared with QM6a. Otherwise, the phenotype was similar to QM6a.

With these female fertile strains in hand, we prepared strains lacking *vel1* in the female fertile background as described above in both mating types [Δ*vel1F1* in MAT1‐1 (abbreviated as V1) and Δ*vel1F2* in MAT1‐2 (V2)]. The phenotype caused by deletion of *vel1* in QM6a as described above was not significantly altered in these strains (data not shown).

**Figure 1:**
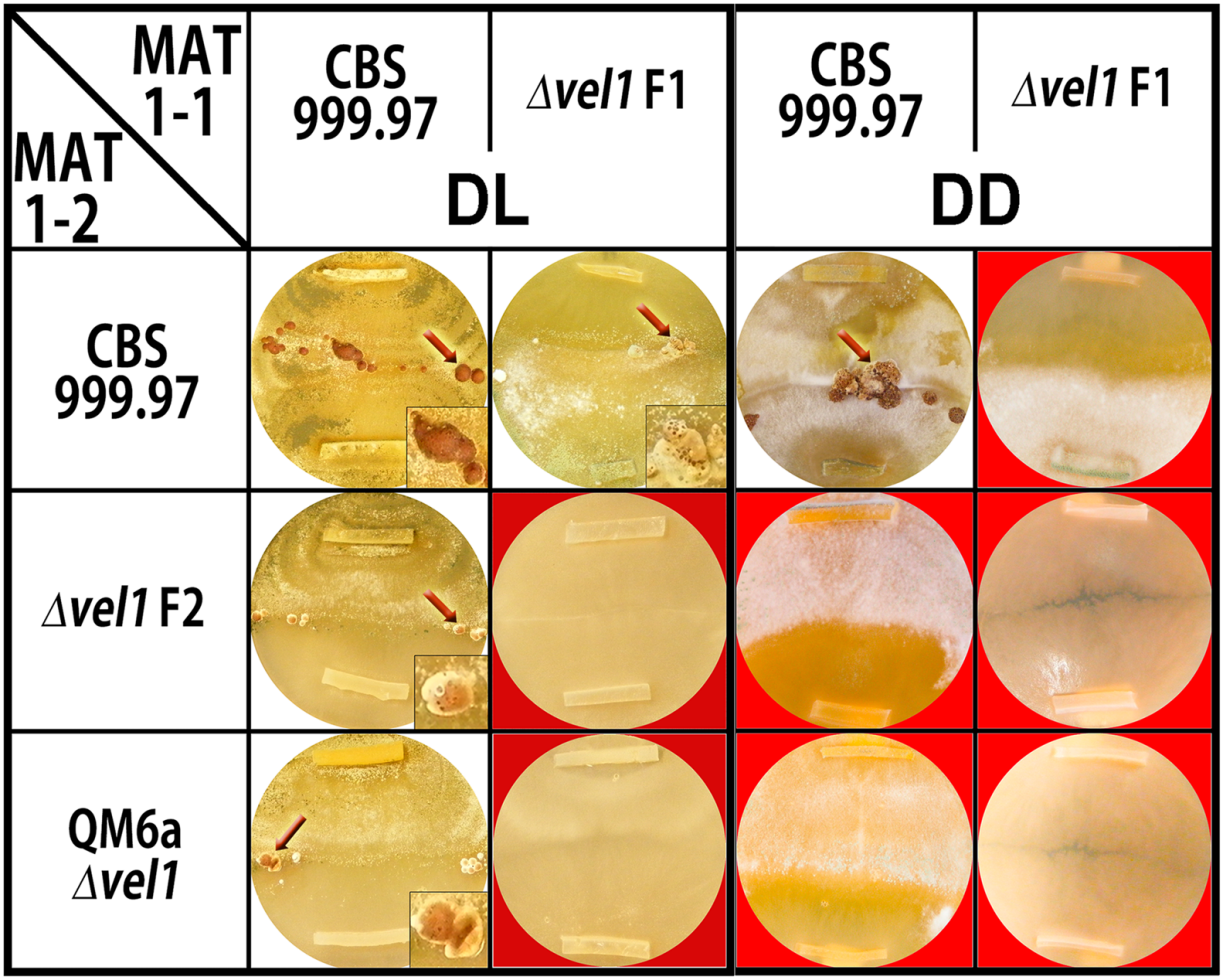
Analysis of sexual development of mutant strains lacking *vel1*. MAT1‐1 strains are inoculated on top; MAT1‐2 strains are inoculated on bottom. Fruiting bodies are indicated with arrows and shown enlarged in the right corner for inoculation in daylight (DL). Fruiting bodies from crosses with strains lacking VEL1 are pale compared with wild‐type crosses. Strains were grown on malt extract agar (MEX) as carbon source in darkness (DD) or daylight (DL), light–dark cycles) at 22°C for 10 days. Red background highlights abolishment of fruiting body formation.

To analyze the role of *vel1* in sexual development, Δ*vel1F1*, Δ*vel1F2* and QM6a Δ*vel1* were crossed with wild‐type strains of CBS999.97 of the corresponding mating type under daylight (cycles of 12 h light–12 h dark) conditions, which are optimal for sexual development in *T. reesei* (Seidl *et al*., 2009; Chen *et al*., 2012). Crosses between the two wild‐type strains resulted in fruiting bodies 7 days after inoculation. However, mating between a wild‐type strain and a Δ*vel1* strain was delayed and only lower numbers of fruiting bodies appeared after 10–12 days (Fig. 1). Moreover, ascosporogenesis was delayed and strongly decreased compared with the wild‐type crosses as ascospores were hardly detectable on the lids of the petri dishes. Progeny of these crosses, which retained the *vel1* deletion, were used as control to confirm that lack of *vel1* causes the observed defects and showed the expected phenotype. Interestingly, the color of the fruiting bodies formed in crosses with *vel1* deletions was somewhat lighter than of those formed in wild‐type crosses. Crosses among two strains, which both had a deletion in *vel1*, failed regardless of the strain background (Fig. 1). The same crossings as described above were repeated in constant darkness, where the wild type forms fruiting bodies with some delay and in lower numbers (Seibel *et al*., 2012a). We found that in every cross in which one partner had lost *vel1*, no fruiting bodies were formed, not even after prolonged incubation (Fig. 1). Hence, VEL1 is essential for sexual development in darkness.

### Female fertility is perturbed in strains lacking VEL1

To determine whether VEL1 is important for male fertility, we used QM6a Δ*vel1*. As this strain is female sterile because of the QM6a background, potential loss of male fertility due to the deletion of *vel1* would result in an inability to mate with even a fully sexual competent mating partner. As crossing of QM6a Δ*vel1* (MAT1‐2) with CBS999.97 (MAT1‐1) was successful in light, albeit with some delay compared with wild‐type crosses (Fig. 1), we conclude that VEL1 is relevant, but not essential for male fertility.

As the presence of VEL1 in at least one mating partner is required for sexual development, we assumed that VEL1 might be involved in regulation of communication between mating partners and/or influence female fertility. Deletion of pheromone receptors leads to female sterility in a mating type‐dependent manner, and mutants of CBS999.97 Δ*hpr1* MAT1‐1 and CBS999.97 Δ*hpr2* MAT1‐2 were found to be female sterile (Seibel *et al*., 2012b). As these strains are still male fertile, they can be used to study the function of VEL1 in female fertility. If VEL1 perturbs female fertility, then sexual crossing with a female sterile partner would not be possible with the respective deletion strains.

**Figure 2:**
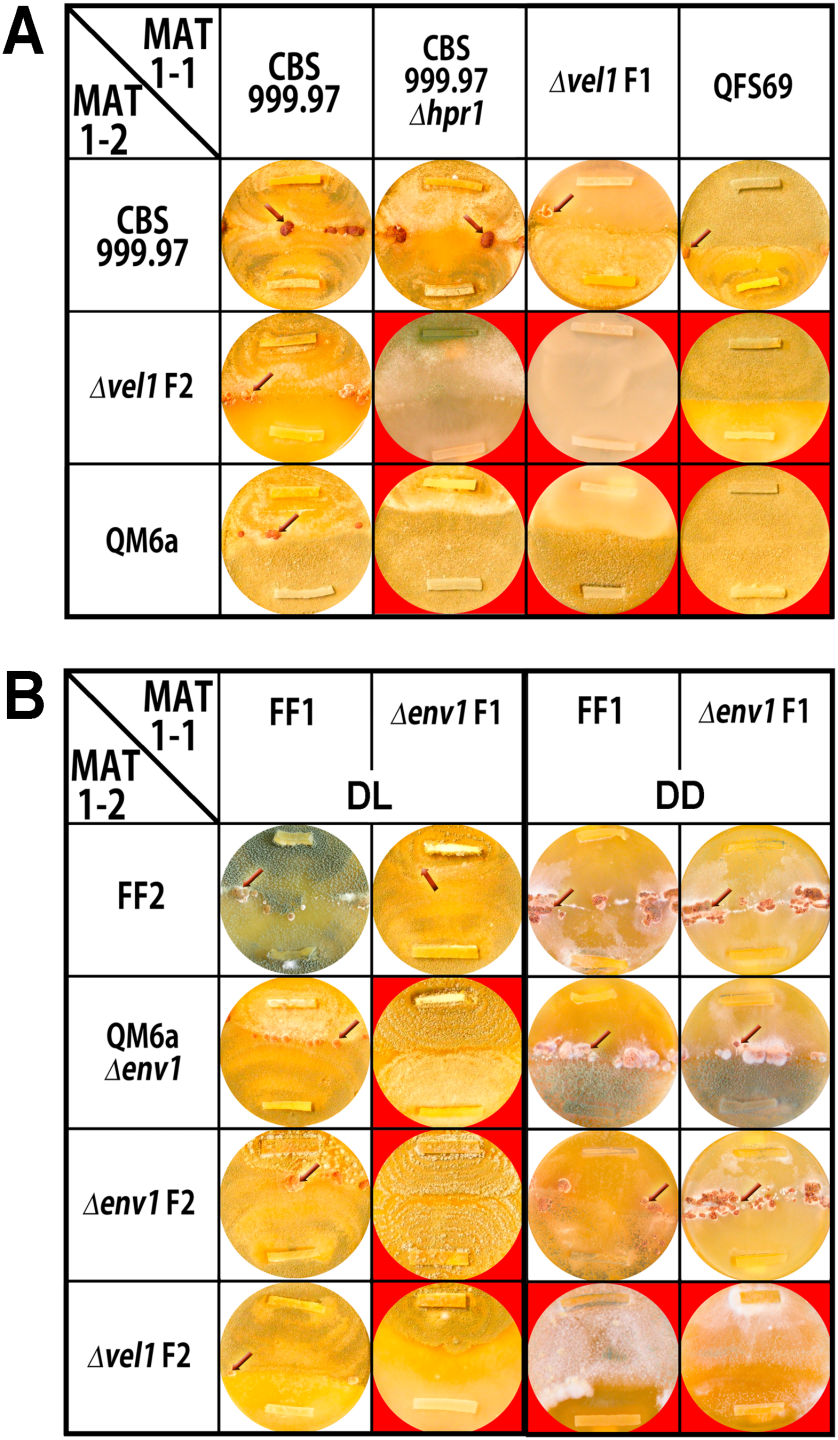
Analysis of female fertility of mutant strains lacking *vel1*. MAT1‐1 strains are inoculated on top; MAT1‐2 strains are inoculated on bottom. Fruiting bodies are indicated with arrows. Strains were grown on malt extract agar (MEX) as carbon source in darkness (DD) or daylight (DL), light–dark cycles) at 22°C for 10 days. Red background highlights abolishment of fruiting body formation.

Fruiting body formation did not occur between CBS999.97 Δ*hpr1* MAT1‐1 (female sterile) and QM6a (female sterile) or Δ*vel1F2*, while a cross between CBS999.97 Δ*hpr1* MAT1‐2 (female fertile) and Δ*vel1F1* resulted in fruiting bodies, which is in agreement with the necessity of VEL1 for female fertility (Fig. 2A). In order to further validate this result, Δ*vel1F2* was crossed with a female sterile derivative of QM6a (QFS69, MAT1‐1), which had been obtained in parallel to FF1 and FF2 upon selection for female sterile strains. Mating failed between these two mating partners, which confirms that VEL1 is essential for female fertility (Fig. 2A). Tests with the other mating types yielded consistent results. Fruiting bodies of *Δvel1* strains were smaller and fewer than in wild‐type crosses. As fruiting body formation does not occur at all upon lack of *vel1* in one mating partner in darkness, we could only investigate these effects upon growth in daylight (12:12 cycles).

As we assumed that mate recognition or sensing might be perturbed upon lack of *vel1*, we tested whether mating would be successful with Δ*env1* strains, which strongly overexpress peptide pheromone precursor genes in light. Lack of ENV1 causes loss of sexual identity by deregulated transcription of pheromone receptors and precursors in light, but not darkness (Seibel *et al*., 2012a). Therefore, we used a Δ*env1* strain with the QM6a background and prepared strains lacking ENV1 in a female fertile background with MAT1‐1 (Δ*env1F1*). In agreement with the female sterility of Δ*env1* strains in light (Seibel *et al*., 2012a), no fruiting bodies emerged in a cross between Δ*vel1F2* and Δ*env1F1* (Fig. 2B). As expected from our experiments described above, also in darkness, no fruiting bodies were formed in crosses between Δ*vel1F* and Δ*env1F* (Fig. 2B). Consequently, the deregulated pheromone system in Δ*env1F* obviously could not overcome a potential sensing defect of Δ*vel1F*.

### Pheromone precursor genes *hpp1* and *ppg1* are differently regulated by VEL1

**Figure 3:**
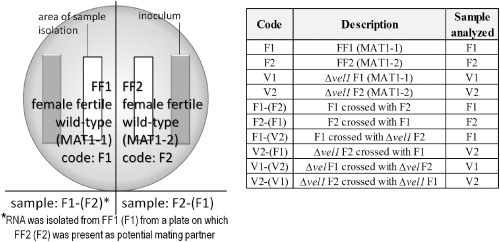
Schematic representation of sample preparation. Samples were harvested separately for both strains present on a plate close to the growth front, but avoiding overlap. Sample codes include the mating partner, which was present on the plate along with the strain from which mycelium was isolated, in brackets.

Several of our results described above indicate a function of VEL1 in environmental sensing and communication with mating partners. We therefore analyzed transcript abundance of pheromone receptor genes and peptide pheromone precursor genes in different mating partner combinations under optimal crossing conditions (12:12 light–dark cycles). Mycelia were consistently harvested upon contact (but not overgrowth) at subjective noon in order to avoid any alterations by circadian rhythms (Fig. 3). Several plates in equal growth stages were pooled and additionally two biological replicates were included. Contamination of total RNA of one sample with that of the respective mating partner on the same plate was analyzed by quantitative polymerase chain reaction (qPCR) from co‐precipitated DNA as described previously (Seibel *et al*., 2012b) and determined to be below 1.02%. This minor contamination did not interfere with reliable analysis of transcript patterns in the results shown. Asexual cultures without mating partners on the same plate were grown in parallel under equal conditions and harvested at the same time points as with sexual development.

**Figure 4:**
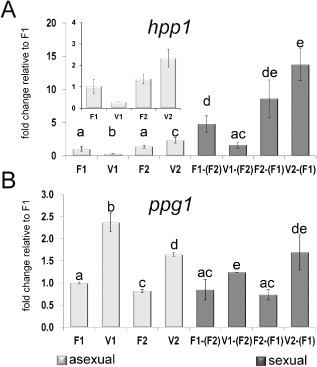
Analysis of pheromone precursor transcript levels. Transcript abundance is shown upon growth on malt extract agar plates at subjective noon as analyzed by qRT‐PCR for both mating types of analyzed strains. Sample codes are summarized in Fig. 6. Different letters indicate significantly different transcript levels. A. Regulation of *hpp1* under asexual conditions in wild type (F1, F2) and strains lacking *vel1* (V1, V2) as well as regulation of *hpp1* upon encounter of the compatible wild‐type mating partner (F1 or F2) under sexual conditions in wild type [F1 crossed with (F2), F2 crossed with (F1)] and strains lacking *vel1* [V1 crossed with (F2), V2 crossed with (F1)]. The insert shows regulation of *hpp1* under asexual conditions. B. Regulation of *ppg1* under asexual conditions in wild type and strains lacking *vel1* as well as regulation of *ppg1* upon encounter of the compatible wild‐type mating partner under sexual conditions in wild type and strains lacking *vel1*. Pooled samples from several plates and two independent biological replicates were considered. Statistical significance of differences was evaluated with the software qbase+ and applying analysis of variance with a *P*‐value threshold of < 0.05. Error bars show standard deviations. Contamination of total RNA of one sample with that of the respective mating partner on the same plate was determined to be below 1.02%.

A significant effect of VEL1 on transcription of the pheromone precursor gene *hpp1* was observed dependent on the mating type. Under asexual conditions, *hpp1* was fivefold downregulated in *Δvel1F1*, whereas the deletion of *vel1* in a MAT1‐2 background (*Δvel1F2*) only had a small influence on *hpp1* transcript levels (Fig. 4A).

A similar positive effect of VEL1 on *hpp1* transcript abundance in strains with a MAT1‐1 background was observed under sexual conditions (Fig. 4A). However, for *Δvel1F2*, the *hpp1* transcript levels showed no significant difference to wild‐type levels.

In contrast to *hpp1*, the pheromone precursor *ppg1* seems to be negatively regulated by VEL1 independent of mating type in asexual conditions (Fig. 4B). *ppg1* transcript levels increased in *Δvel1* strains in both mating types (Fig. 4B; 2.0 fold ± 0.05 in MAT1‐2, 2.37 fold ± 0.26 in MAT1‐1). The same regulative pattern was observed in sexual conditions (Fig. 4B). We conclude that VEL1 differentially regulates both pheromone precursor genes in a mating type‐dependent manner.

### VEL1 is a positive regulator for transcription of pheromone receptors

**Figure 5:**
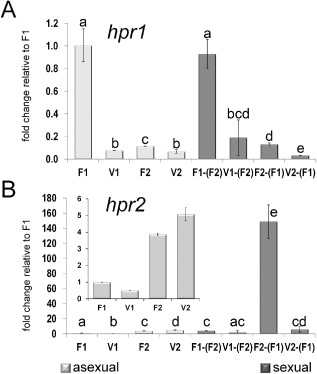
Analysis of pheromone receptor transcript levels. Transcript abundance is shown upon growth on malt extract agar plates at subjective noon as analyzed by qRT‐PCR for both mating types of analyzed strains. Sample codes are summarized in Fig. 6. Different letters indicate significantly different transcript levels. A. Regulation of *hpr1* under asexual conditions in wild type (F1, F2) and strains lacking *vel1* (V1, V2) as well as regulation of *hpr1* upon encounter of the compatible wild‐type mating partner (F1 or F2) under sexual conditions in wild type [F1 crossed with (F2), F2 crossed with (F1)] and strains lacking *vel1* [V1 crossed with (F2), V2 crossed with (F1)]. B. Regulation of *hpr2* under asexual conditions in wild type and strains lacking *vel1* as well as regulation of *hpr2* upon encounter of the compatible wild‐type mating partner under sexual conditions in wild type and strains lacking *vel1*. The insert shows regulation of *hpr1* under asexual conditions. Pooled samples from several plates and two independent biological replicates were considered. Statistical significance of differences was evaluated with the software qbase+ and applying analysis of variance with a *P*‐value threshold of < 0.05. Error bars show standard deviations. Contamination of total RNA of one sample with that of the respective mating partner on the same plate was determined to be below 1.02%.

First, we checked mating type‐specific regulation in order to compare our data with earlier results. Both pheromone receptor transcripts (*hpr1* and *hpr2*) show a mating type‐dependent regulation in the wild‐type strains FF1 or FF2 (Fig. 5). Transcription of *hpr1* is enhanced in MAT1‐1, whereas *hpr2* is strongly upregulated in MAT1‐2 background even in asexual conditions. A similar pattern was previously observed for pheromone receptor mutants in CBS999.97 (Seibel *et al*., 2012b). Hence, also considering transcription patterns of pheromone precursor genes, the pheromone system of *T. reesei* is consistently regulated in strains with fertile QM6a background (FF1 and FF2) and CBS999.97 strains.

Under asexual conditions, VEL1 positively regulates *hpr1* in MAT1‐1, with transcript levels of *hpr1* dropping to MAT1‐2 levels in the deletion mutant. In MAT1‐2, only a minor positive effect of VEL1 was detected (Fig. 5A). Also upon encounter of a mating partner (sexual conditions), *hpr1* levels were decreased in *Δvel1F1* compared with wild type and drop to MAT1‐2 levels in this strain. In *Δvel1F2* (MAT1‐2), transcript abundance drops even further below wild‐type levels (Fig. 5A).

*Hpr2* was found to be downregulated in *Δvel1F1* compared with wild type in MAT1‐1, whereas only a small alteration was observed in MAT1‐2 (Fig. 5B) under asexual conditions. A much stronger mating type‐dependent regulation of *hpr2* was observed under sexual conditions. While in MAT1‐1 no significant difference was observed in *hpr2* transcript levels between wild type and *Δvel1F1* (both in the presence of wild type)*,* we found a considerable decrease in transcript abundance of this gene upon deletion of *vel1* in MAT1‐2 to levels around those in MAT1‐1 (Fig. 5B).

In summary, VEL1 is a positive regulator important for mating type‐specific regulation of pheromone receptor genes with substantial effects on *hpr1* and *hpr2* transcript abundance, especially upon encounter of a mating partner and in their cognate mating type.

### The mating partner influences regulation of the pheromone system in Δvel1F

**Figure 6:**
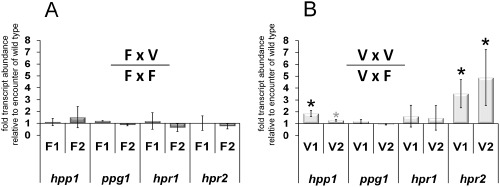
Comparison of transcript levels in wild‐type or *vel1* mutants upon encounter of wild‐type or mutant mating partners. Transcript abundance is shown upon growth on malt extract agar plates at subjective noon as analyzed by qRT‐PCR for both mating types of analyzed strains. The results for every datapoint represent a comparison between encounter of the mutant strain (V) and the wild‐type strain (F). Therefore, whenever the result shown in one column including the error bar shows an increase above 1, the result is statistically significant. A. Regulation of *hpp1*, *ppg1*, *hpr1* and *hpr2* in wild type (F1 or F2) if Δ*vel1F* is the mating partner compared with transcript levels in wild type if the compatible wild type is the mating partner [e.g. ‘F1’ here represents (FF1xΔ*vel1F2*)/(FF1xFF2)]. B. Regulation of *hpp1*, *ppg1*, *hpr1* and *hpr2* in Δ*vel1F* (V1 or V2) if Δ*vel1F* is the mating partner compared with transcript levels in Δ*vel1F* if the compatible wild type is the mating partner [e.g. ‘V1’ here represents (V1xΔ*vel1F2*)/(V1xFF2)]. Asterisks indicate significant difference between encounter of the wild‐type and encounter of the *vel1* mutant strain on the plate. For *hpp1* in Δ*vel1F2* (V2), the gray asterisk indicates only a minor effect. Pooled samples from several plates and two independent biological replicates were considered. Statistical significance of differences was evaluated with the software qbase+ and applying analysis of variance with a *P*‐value threshold of < 0.05. Error bars show standard deviations.

Analysis of the function of VEL1 in transcriptional regulation of the pheromone system clearly indicated a function in mating partner communication for VEL1. Consequently, we investigated the effect of different partners in sexual development on transcript levels. Hence, in addition to the wild type, we used the corresponding Δ*vel1F* strain as mating partner and analyzed transcript levels of pheromone precursor and pheromone receptor genes. Fig. 6 compares fold changes of transcript levels if a *vel1* mutant was the partner instead of wild type for *hpp1*, *ppg1*, *hpr1* and *hpr2* in both mating types. Values significantly different from 1 (1 = regulation independent of partner) represent an altered response to Δ*vel1F* compared with the response to wild type.

For crosses of wild‐type strains FF1 and FF2, we did not see any significant difference in expression of pheromone receptor or precursor genes regardless if the compatible wild type or a *vel1* mutant was the mating partner (Fig. 6A). Regarding Δ*vel1F* strains, no significant differential partner effects were found for *ppg1* and *hpr1*, the cognate pheromone precursor and receptor of the MAT1‐1 mating type.

Interestingly, we found lower transcript levels of *hpp1* in Δ*vel1F1* (MAT1‐1) if the wild‐type FF2 was the partner than if Δ*vel1F2* was the partner. With Δ*vel1F2* (MAT1‐2), no such partner‐dependent effect occurred for *hpp1* (Fig. 6B). Hence, it appears that *hpp1* levels are not only influenced by the mating type of a given strain, but also by the mating partner and that the underlying mechanism is altered in the absence of VEL1. As *hpp1* is not the pheromone of MAT1‐1, its downregulation upon encounter of a mating partner is explainable and does not happen equally in a VEL1 mutant.

For *hpr2*, the cognate pheromone receptor of MAT1‐2, we found a similar trend. Transcript abundance for *hpr2* in Δ*vel1F2* was clearly higher if Δ*vel1F1* was the partner compared with wild type as a partner. The same situation occurs if Δ*vel1F1* senses Δ*vel1F2* compared with sensing of wild type (Fig. 6B). Keeping in mind that transcript levels of *hpr2* in Δ*vel1F2* were considerably lower than in wild‐type FF2 in the presence of a mating partner [Fig. 5B, F2‐(F1) compared with V2‐(F1)], the increased levels seen in the presence of Δ*vel1F1*, but not wild‐type as the partner, reflect a situation closer to the reaction of the wild‐type strains to one lacking *vel1*. This result hints to a potentially perturbed feedback, which would likely involve pheromone signal perception in the other mating type.

### VEL1 is involved in partner‐dependent *mat1‐2‐1* transcription

*hpp1* and *hpr2,* which appear to be subject to feedback regulation, encode the cognate pheromone precursor and pheromone receptor of mating type MAT1‐2.

**Figure 7:**
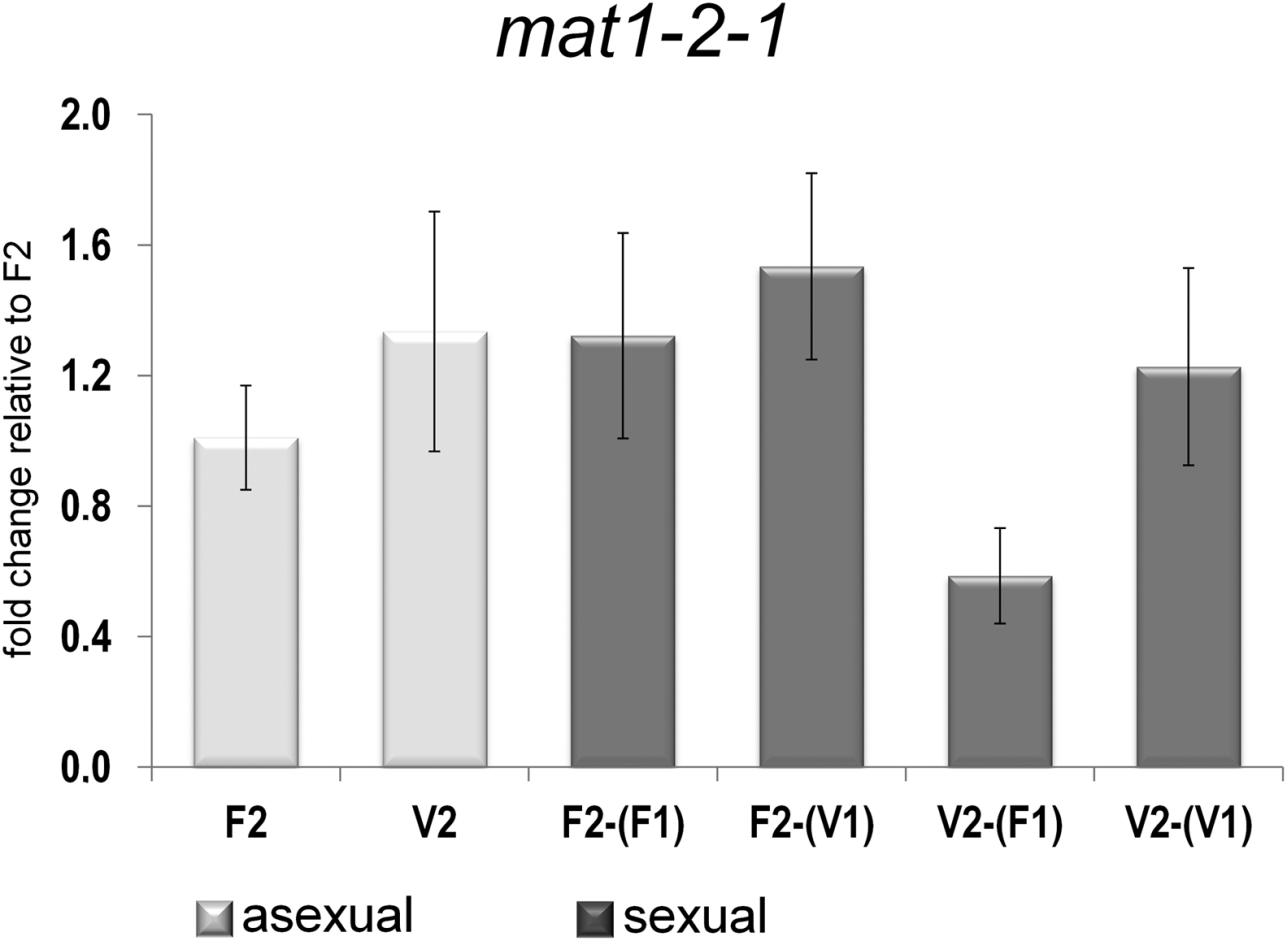
Analysis of *mat1‐2‐1* transcript levels. Transcript abundance is shown upon growth on malt extract agar plates at subjective noon as analyzed by qRT‐PCR for both mating types of analyzed strains. Sample codes are summarized in Fig. 6. Regulation of *mat1‐2‐1* under asexual conditions in wild type (F2) and Δ*vel1* *F* *2* (V2) along with regulation of *mat1‐2‐1* upon encounter of the compatible wild‐type (F1) or Δ*vel1* *F* *1* (V1) mating partner under sexual conditions in wild type [F2 crossed with (F1)] and strains lacking *vel1* [V2 crossed with (F1) or V2 crossed with (V1)]. Transcript levels between sexual and asexual conditions are not significantly different for the wild type [F2 vs. F2‐(F1)]. Only in Δ*vel1* *F* *2* upon encounter of the wild type [V2‐(F1)] *mat1‐2‐1* is significantly downregulated compared with all other samples. Pooled samples from several plates and two independent biological replicates were considered. Statistical significance of differences was evaluated with the software qbase+ and applying analysis of variance with a *P*‐value threshold of < 0.05. Error bars show standard deviations. Contamination of total RNA of one sample with that of the respective mating partner on the same plate was determined to be below 1.02%.

This prompted us to screen for VEL1 regulation and partner effects on the respective mating type gene *mat1‐2‐1*. Under asexual conditions, *mat1‐2‐1* is not regulated by VEL1 (Fig. 7). However, in sexual crosses, transcript levels of *mat1‐2‐1* in Δ*vel1F2* were at wild‐type levels if Δ*vel1F1* was the crossing partner, but clearly decreased if wild‐type FF2 was the partner (to 44% of wild type; Fig. 7). Comparable with regulation of *hpp1* and *hpr2*, the response of Δ*vel1F2* to wild type was altered compared with encounter of another strain lacking *vel1*. Consequently, VEL1 is involved in partner‐dependent regulation of mating type‐specific gene expression.

### VEL1 regulates communication between mating partners by secondary metabolites

The partner effects we observed did suggest an involvement of pheromone regulation in the response of wild‐type strains and those lacking *vel1* to their partner. In wild type, pheromone and receptor expression appears to be well balanced, but in the absence of *vel1*, this balance is perturbed. Additionally, the strongly upregulated pheromone expression in Δ*env1*F could not compensate for the assumed sensing defect because of decreased transcript levels of pheromone receptors in Δ*vel1*F strains. Consequently, we assumed that partner sensing as regulated by VEL1 is not limited to pheromones. Because of the well studied function of VELVET proteins in secondary metabolism, we figured that secreted metabolites might contribute to an altered response to an encountered mating partner. As we assume that these metabolites represent secondary metabolites, which are not required for growth or metabolism, we will refer to these compounds as such.

For that purpose, we applied semi‐quantitative high‐performance thin layer chromatography (HPTLC) using different visualization techniques to detect different substance classes. As secondary metabolism in *T. reesei* has hardly been studied yet, identity of the regulated compounds remains to be elucidated and work on this topic is in progress. In the following, we show selected results from the visualization techniques that most clearly showed the differences we found. Thereby, the different techniques are specific for different compounds. Agar without mycelium, but otherwise treated equally, was used as control. Pooled samples and two biological replicates were considered for interpretation of results. We want to note here that in some cases, compounds detected in the control sample are apparently degraded to different extent by different strains. Such degradation is not attributed to secondary metabolism, but rather reflects differential enzymatic activity between strains and is hence considered in our interpretation.

**Figure 8:**
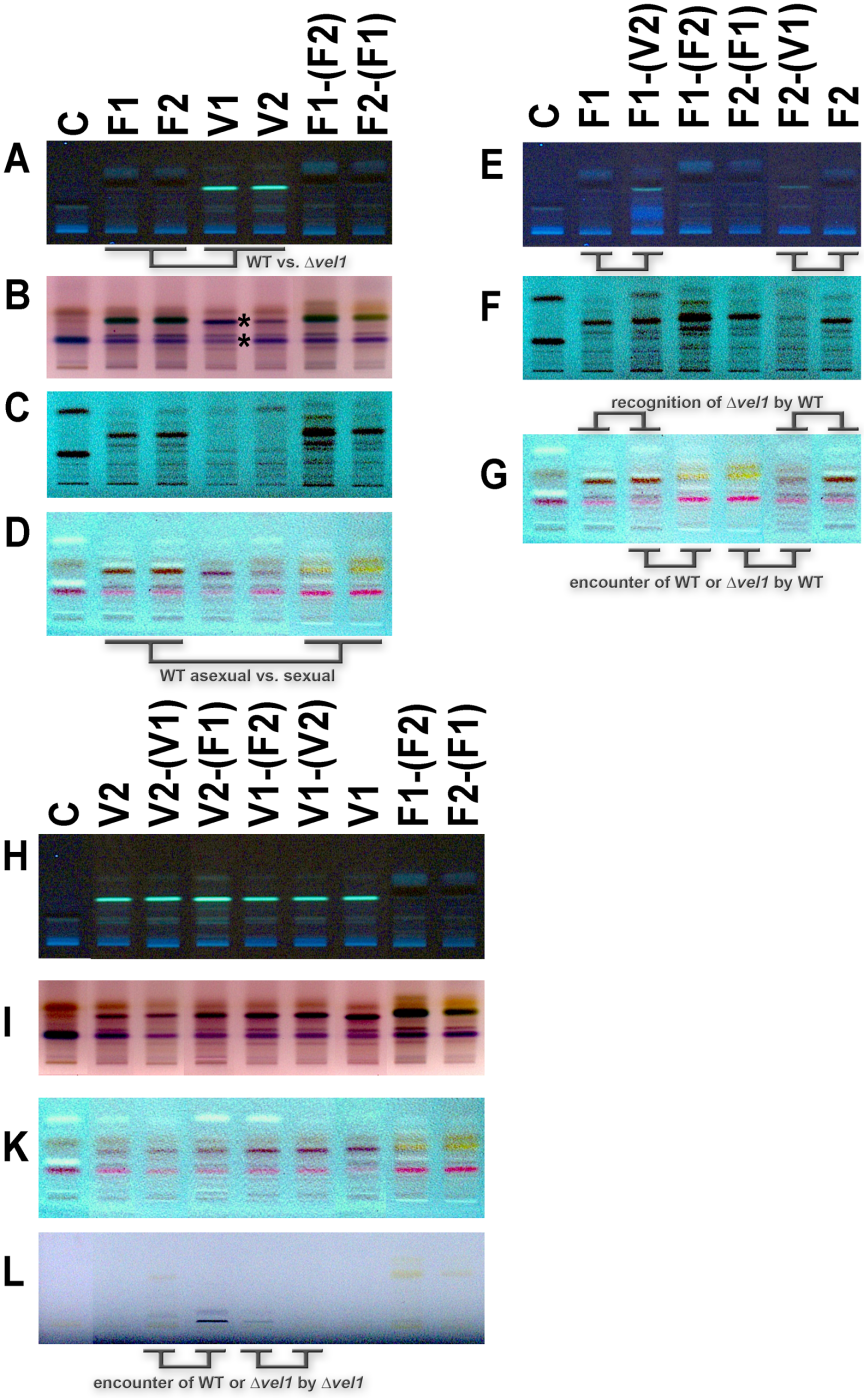
Secondary metabolite patterns of *T* *richoderma reesei* under asexual and sexual conditions. Sample codes are summarized in Fig. 6. A–D. Secondary metabolite patterns of wild‐type (WT) (F1, F2) and *vel1* deletion strains (V1, V2) in both mating types and of WT upon encounter of a compatible WT mating partner [F1‐(F2) or F2‐(F1)]. Asterisks in B show the bands differing between *Δvel1F* stains in different mating types. E–G. Secondary metabolite patterns of WT under asexual conditions (F1 or F2) and upon response to a compatible WT strain [F1‐(F2) or F2‐(F1)] or to a strain lacking *vel1* [(F1‐(V2) or F2‐(V1)]. H–L. Secondary metabolite patterns of *Δvel1F* under asexual conditions (V1 or V2) and upon response to a compatible WT strain [V1‐(F2) or V2‐(F1)] or to a strain lacking *vel1* [(V1‐(V2) or V2‐(V1)]. Growth medium treated equally was used as control (‘C’). The different panels show different visualization techniques, which allow for detection of different substance classes in the sample. (A, E, H) Remission at 366 nm; (B, I) derivatized with anisaldehyde – sulfuric acid, transmission, visual light; (C, F) remission at 254 nm; (D, G, K) derivatized with anisaldehyde – sulfuric acid, remission at 254 nm; (L) remission, transmission at visual light. In order to enable optimal comparison between samples, pictures were reassembled. All samples shown in one panel are combined from the same HPTLC plate. All picture analyses were performed using the analysis software visionCATS 1.4.14017.1 (CAMAG) on the whole picture of the respective plate. Two biological replicates were made and considered for interpretation. The plates with original loading order are provided as Supporting Information material (Supporting Information Fig. S2) for comparison.

HPTLC revealed that secondary metabolites secreted by FF1 or FF2 in the absence of a mating partner were similar (Fig. 8A–C). Δ*vel1F* strains showed altered secondary metabolite patterns compared with FF1 and FF2, which confirms a function in secondary metabolism in *T. reesei* as well. However, in contrast to what could be expected from other species, secretion of secondary metabolites was not simply diminished, but for some metabolites even increased in these mutants (Fig. 8A) and decreased for others (Fig. 8B and C).

In order to evaluate if partner effects as seen in the transcript analysis also occurred for secondary metabolite patterns, we analyzed different partner combinations. The secondary metabolite pattern of wild‐type strains FF1 and FF2 clearly and consistently changed if a mating partner was present (Fig. 8C and D). Consequently, communication between mating partners or generally upon encounter of another fungus in the environment involves secondary metabolites in *T. reesei* as well. The derivatization method we used (p‐anisaldehyde/sulfuric acid) allows for detection of phenols, sugars, steroids and terpenes. Hence, an involvement of an isoprenoid compound related to trisporic acid, which has pheromone function in zygomycetes (Schimek and Wostemeyer, 2009), could contribute to this response in *T. reesei*.

We also tested the response of FF1 and FF2 to the respective compatible strains lacking *vel1* (Δ*vel1F1* and Δ*vel1F2*). Clear differences in metabolite patterns were observed for both FF1 and FF2 between encountering wild type or a mutant lacking *vel1* (Fig. 8E–G). Although specific differences were also found (Fig. 8E), for most visualization techniques, the patterns secreted by FF1 and FF2 rather resemble the situation of those strains growing alone on the plate (Fig. 8F and G). These results suggest that recognition is hampered if *vel1* is lacking in the partner's genome. Additionally, *vel1* may be responsible for production of a metabolite (or metabolite combination) that elicits a specific response upon encounter.

In Δ*vel1F1* and Δ*vel1F2*, the pattern observed upon encounter of a potential mating partner differed from that in the wild type (Fig. 8H). Our transcript analysis revealed that strains lacking *vel1* respond differently depending on whether they encounter wild type or Δ*vel1F* strains. For most metabolites, as reflected by different visualization techniques, only minor alterations between presence of wild type or a strain lacking *vel1* were detected (Fig. 8H, I and K). Degradation of a compound in the medium upon encounter of strains lacking *vel1* in contrast to wild type (uppermost band on Fig. 8K) suggests modulated enzymatic activities. Nevertheless, Δ*vel1F1* and in lower amounts also Δ*vel1F2* produce a secondary metabolite upon encounter of wild type but not *vel1* mutants, which is not detected in wild type (Fig. 8L).

## Discussion

Sexual development is of utmost importance in the physiology and evolution of fungi. With *T. reesei,* application of sexual crossing for industrial strain improvement adds another aspect for investigation of development. Thereby, communication between fungi prior to initiation of mating is crucial for the success of crossing.

Our study revealed a defect in female fertility in light, in mating in darkness as well as in conidiation to be caused by the lack of VEL1. While defects in conidiation upon deletion of *vel1* have been observed in other fungi (Kim *et al*., 2002; Mukherjee and Kenerley, 2010; Kopke *et al*., 2013), a function in female fertility specifically in light has not been reported for VEL1 homologs before. In *A. nidulans*, VeA plays a crucial role in balancing asexual and sexual development (Kim *et al*., 2002). Apparently, the function of VEL1 in *T. reesei* targets both processes: upon deletion of *vel1*, conidiation is abolished and sexual development is severely perturbed. Similar defects in asexual development were also observed in *T. virens* strains lacking *vel1* (Mukherjee and Kenerley, 2010).

**Figure 9:**
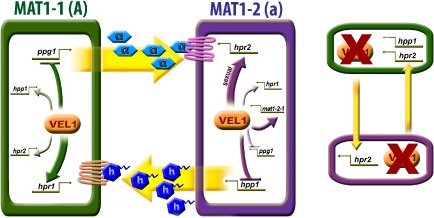
Model of VEL1 regulation of the pheromone system. VEL1 is involved in regulation of both pheromone receptors and pheromone precursors in both mating types under sexual and asexual conditions. The most striking regulation is thereby exerted on the pheromone receptors in their cognate mating types (*hpr1* in MAT1‐1 and *hpr2* in MAT1‐2). Considering the function of pheromone receptors in their cognate mating type in female fertility, this regulation is likely to contribute to female fertility of *vel1* mutants. Strains lacking *vel1* react to the presence of another *vel1* mutant by upregulation of *hpr2* in MAT1‐2 and of *hpr2* and *hpp1* in MAT1‐1 compared with encounter of a wild‐type strain, where this response does not occur. Only effects with statistically significant regulation in the respective cognate mating type are shown in the scheme with arrows for positive and plungers for negative regulation.

Interestingly, although VEL1 was found to be involved in regulation of the pheromone system under asexual conditions as well, the most striking regulation was detected for the pheromone receptors in their cognate mating type under sexual conditions (Fig. 9). *Hpr1* transcript levels are 5‐fold decreased in MAT1‐1 and *hpr2* is 25‐fold downregulated in MAT1‐2 under sexual conditions if *vel1* is deleted. As these pheromone receptors in their cognate mating type are important for female fertility (Seibel *et al*., 2012b), it seems possible that this regulation is responsible for the defect in female fertility of Δ*vel1* strains or at least contributes to this defect.

Studying partner effects in regulation of transcript levels of pheromone precursor and receptor genes, we found that strains lacking *vel1* respond differently to the presence of a *vel1* deletion strain than to wild‐type strains in sexual crosses. This different response is restricted to *hpp1* and *hpr2*, the pheromone precursor and receptor genes associated with the MAT1‐2 mating type. Accordingly, a similar partner effect was also observed for *mat1‐2‐1*, the mating type gene of the MAT1‐2 mating type, upon lack of *vel1*. *mat1‐2‐1* is also regulated by VEL1 under sexual conditions, hence supporting a function of VEL1 in mating type‐dependent regulation of sexual development.

One explanation of the partner effect we saw would be the operation of a feedback mechanism, which responds to altered levels of pheromones. These pheromones are assumed to be secreted by Δ*vel1F* strains based on their altered transcript levels and are then sensed in the environment as well as by the fungus secreting them itself. Sensing and secreting the same signaling molecule is a ubiquitous mechanism for communication among fungi (Youk and Lim, 2014) and reflects an adaptation to a changing environment. Feedback regulation of pheromone expression has been shown previously and involves heterotrimeric G‐protein signaling (particularly also through beta and gamma subunits) and mitogen‐activated protein (MAP) kinase pathways (Feng and Davis, 2000). It has been shown in *A. nidulans* that the MAP kinase pathway impacting development and secondary metabolite production targets VeA (Bayram *et al*., 2012).

The G‐protein pathway is known to sense pheromones and to adjust downstream gene regulation. This is in accordance with our findings that lack of the G‐protein beta subunit decreases *hpp1* transcript levels and abolishes ascosporogenesis in *T. reesei* (Tisch *et al*., 2011b). As the presence of pheromones in the environment is sensed by pheromone sensors, which are not transcribed in a strictly mating type‐dependent manner in *T. reesei* (Seibel *et al*., 2012b), a feedback mechanism is conceivable also in this fungus. However, our analysis of partner effects with strains lacking ENV1, in which the pheromone system is more strongly deregulated than in Δ*vel1F* (Seibel *et al*., 2012a), did not show significant differences upon encounter of different strains. This led us to assume that pheromone regulation alone is unlikely to cause this phenomenon.

A connection between secondary metabolism and development is well known in fungi (Kück and Böhm, 2013). We were hence interested whether the function of VELVET proteins in fungi is involved in mating partner recognition in *T. reesei*. Hardly anything is currently known on secondary metabolites secreted by *T. reesei* (Blumenthal, 2004; Lehner *et al*., 2013) although its genome comprises numerous genes associated with secondary metabolism (Mukherjee *et al*., 2012). Harmful compounds are not known to be secreted by *T. reesei* and hence it achieved GRAS (generally regarded as safe) status by the Food and Drug Administration (21 Code of Federal Regulations §184,1250). However, production of trichodermin (Watts *et al*., 1988) and the peptide antibiotic paracelsin (Brückner *et al*., 1984) was reported.

**Figure 10:**
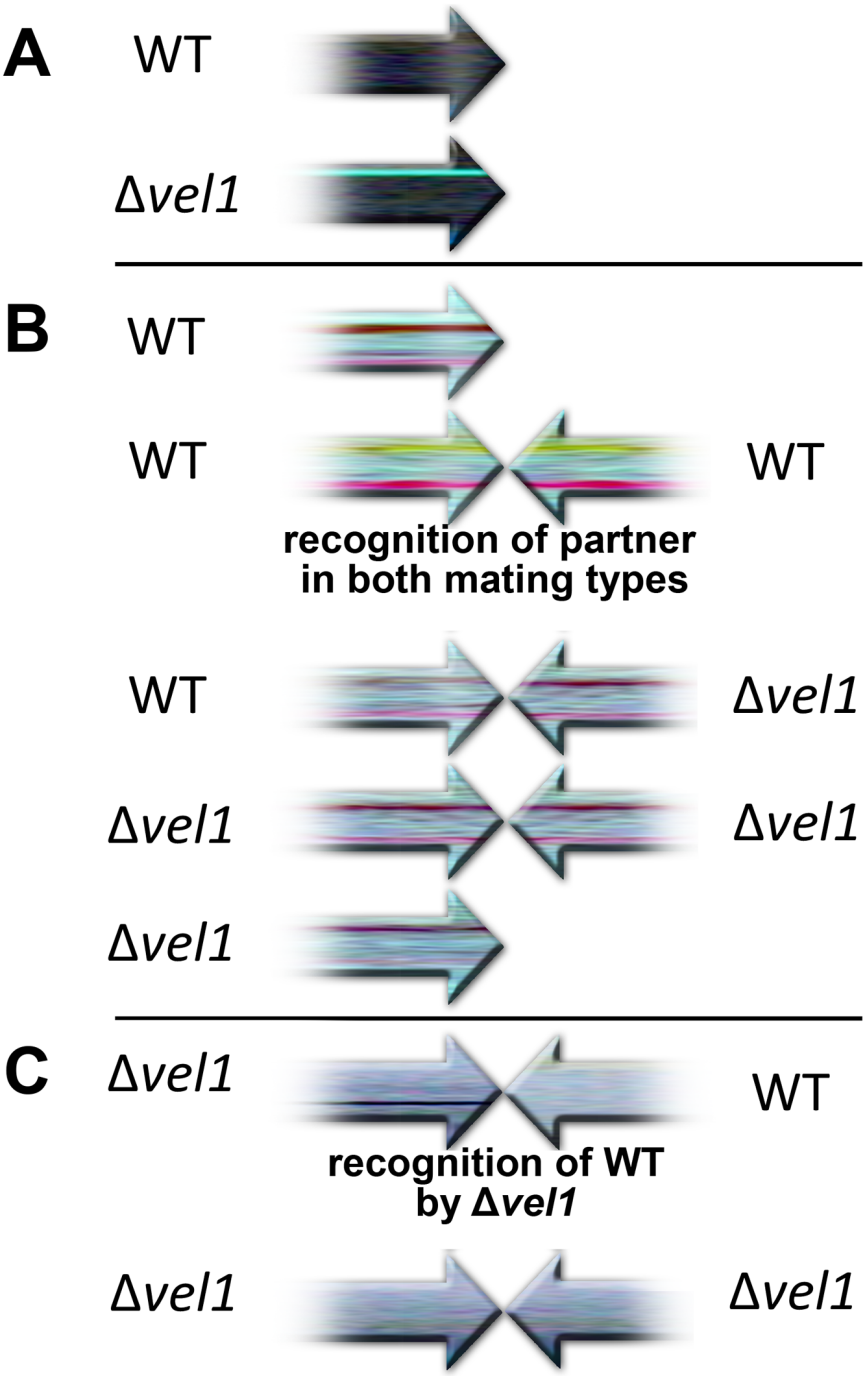
Schematic representation of partner effects in wild‐type (WT) and Δ*vel1*. Metabolite patterns observed in HPTLC are shown as arrows, which are meant to show mutual influence on metabolite patterns. Opposing arrows represent sexual conditions with patterns of both partners on the plates. A. Different metabolite patterns in WT and Δ*vel1* indicate that VEL1 is involved in regulation of secondary metabolism (Fig. 8A; remission at 366 nm). B. WT recognizes the presence of a WT partner on the plate as reflected in altered metabolite patterns compared with growth alone. Presence of Δ*vel1* causes hardly any response by the WT, because metabolite patterns show only minor changes. The same is the case for two Δ*vel1* mutants on the plate (Fig. 8D, G and K; derivatized with anisaldehyde – sulfuric acid, remission at 254 nm). C. Δ*vel1* recognizes WT and alters secondary metabolite production. This does not occur in the presence of another Δ*vel1* strain (Fig. 8L; remission, transmission at visual light). Mycelia were harvested individually as shown in Fig. 3. Bands from the visualization technique, which shows the effect most clearly, were used for the scheme.

As expected, VEL1 was found to be involved in regulation of secondary metabolism as well. Altered secondary metabolite secretion in wild‐type and Δ*vel1* strains is summarized in Fig. 10 along with partner effects upon recognition of wild type or mutant on the plate. Strains lacking VEL1 secrete a different set of metabolites as the wild type already when grown alone on a plate (Fig. 10A). Interestingly, we found that the wild type clearly responds to encounter of another fungus on the plate with altered secondary metabolite profiles. This pattern is again changed if a strain lacking *vel1* is the partner (Fig. 10B), and also Δ*vel1F* reacts differently to wild type or mutant (Fig. 10C). Currently, the nature of these metabolites is not known, but under investigation in our lab.

While extracellular metabolites including oxylipins are known to coordinate a switch between asexual and sexual development (summarized in Christensen and Kolomiets, 2011; Leeder *et al*., 2011), to the best of our knowledge, effects similar to those we observed have not been reported so far from other fungi. However, such effects are to some extent reminiscent of quorum sensing. Additionally, subtle differences in band intensity suggest that the mating type of stains lacking *vel1* influences secondary metabolite production (Fig. 8B). As the lower of the two differing bands appears in the control as well, altered enzymatic activity for degradation of this compound in these strains is likely. Alternatively, true secondary metabolites at this position might have been masked by the background of the medium. Nevertheless, further analyses will be necessary to substantiate this difference.

Quorum sensing has been studied in *Aspergillus flavus* and is reported to involve oxylipin signaling (Tsitsigiannis *et al*., 2004; 2005). Oxylipin signaling has been suggested to be involved in injury response in *Trichoderma atroviride* based on transcriptome data (Hernandez‐Onate *et al*., 2012). In *A. flavus*, this mechanism is perturbed if *veA* or *laeA* is deleted (Amaike and Keller, 2009; Amare and Keller, 2014). However, the specific sensing of a wild‐type strain versus a mutant strain clearly goes beyond quorum sensing, but indicates that VELVET contributes to transmission/interpretation of a more sophisticated communication between fungi. In‐depth studies of secondary metabolism in *T. reesei* are in progress and will reveal whether oxylipin signaling plays a role in this communication.

In summary, *T. reesei* applies a complex chemical language for communication with fungi in their environment, in which *vel1* plays a role in both sending and receiving signals. Hence, the regulation of secondary metabolites by VEL1 goes beyond an involvement in simple defense mechanisms, but is responsible for a sophisticated and fine‐tuned chemical communication.

## Experimental procedures

### Microbial strains and culture conditions

*Trichoderma reesei* (*H. jecorina*) QM6a wild‐type strain (ATCC 13631) was used as the parental strain to construct deletions of *Δvel1*. CBS999.97 MAT1‐1 and MAT1‐2 strains and several other wild‐type and mutant strains from different sources were included in this study to set up informative crosses (Table 1). Female fertile strains FF1 and FF2 with QM6a background were prepared as described previously (Schuster *et al*., 2012), but with 10 instead of 5 crosses.

**Table 1:** Strains used in this study

Strain	Code	Characteristics	Source/reference
QM6a		Wild‐type MAT1‐2	Martinez *et al*. (2008)
CBS999.97 MAT1‐1		Wild‐type MAT1‐1	Seidl *et al*. (2009)
CBS999.97 MAT1‐2		Wild‐type MAT1‐2	Seidl *et al*. (2009)
CBS999.97 MAT1‐1 Δ*hpr1*		Δ*hpr1*::hph+ MAT1‐1	Seibel *et al*. (2012b)
CBS999.97 MAT1‐2 Δ*hpr1*		Δ*hpr1*::hph+ MAT1‐2	Seibel *et al*. (2012b)
FF1	F1	Female fertile derivative of QM6a MAT1‐1	This study
FF2	F2	Female fertile derivative of QM6a MAT1‐2	This study
QFS69 MAT1‐1		Female sterile derivative of QM6a MAT1‐1	This study
QM6a Δ*vel1*		Δ*vel1*::*amds* ^+^ MAT1‐2, female sterile background (QM6a)	This study
QM6a Δ*env1*		Δ*env1*::*hph* ^+^ MAT1‐2, female sterile background (QM6a)	This study
Δ*vel1*F1	V1	Δ*vel1*::*amds* ^+^ MAT1‐1, female fertile background	This study
Δ*vel1*F2	V2	Δ*vel1*::*amds* ^+^ MAT1‐2, female fertile background	This study
Δ*env1*F1	E1	Δ*env1*::*hph* ^+^ MAT1‐1, female fertile background	This study
Δ*env1*F2	E2	Δ*env1*::*hph* ^+^ MAT1‐2, female fertile background	This study

Propagation of strains was performed on 3% (w/v) malt extract agar (Merck, Darmstadt, Germany). Crossing experiments were made on 2% (w/v) malt extract agar at 22°C in daylight (cycles of 12 h light–12 h dark) or constant darkness. Therefore, strains were grown on opposite sides of petri dishes and were evaluated for fruiting body formation and ascospore discharge 7 and 20 days after inoculation respectively. For evaluation of conidiation in QM6a Δ*vel*1, Mandels–Andreotti (MA) medium (Mandels and Andreotti, 1978) supplemented with 1% (w/v) carbon source and 0.1% (w/v) peptone (Merck) to induce germination was used. D‐galactose (Sigma Aldrich, St. Louis, USA), D‐sorbitol (ALFA AESAR, Karlsruhe, Germany), D‐mannitol (Fluka, St. Gallen, Switzerland), D‐arabinose (Sigma Aldrich), meso‐erythritol (ALFA AESAR) and γ‐amino butyric acid (GABA) (Sigma Aldrich) or D(+)‐xylose (Sigma Aldrich) were used as carbon sources in sporulation assays.

For transcriptional analysis, the strains were pre‐cultured on 3% (w/v) malt extract agar in constant darkness for at least 3 days. Inoculation of strains was performed on 2% (w/v) malt extract agar plates covered with cellophane to facilitate harvesting. To study gene expression in the course of sexual development, the strains were inoculated on both sides of the petri dishes and the mycelia of the partner strains were harvested at the stage of contact (3 days after inoculation). As the mutant strains have growth defects compared with wild‐type strains, they were inoculated in different distances from the opposite mating strain on petri dishes in order to obtain the contact stage of mycelia for the mutant strains at consistent times. Five plates at the stage of contact were pooled and at least two biological replicates were used for quantitative reverse transcription polymerase chain reaction (qRT‐PCR) analysis. In order to compare the obtained data with asexual growth, strains were grown alone on plates and the mycelia were harvested at the time points corresponding to the contact of strains in sexual development. The strains were grown under cycles of 12 h light–12 h darkness at 22°C (1800 lux).

For cellulase screening, strains were grown on plates with MA medium containing 1% (w/v) carboxymethylcellulose (CMC) (Sigma Aldrich) and after 2 days were stained with Congo red (0.1% w/v solution in water) (Roth, Karlsruhe, Germany), which stains cellulose (Carder, 1986). After washing of the plate with 1 M NaCl, halos indicate cellulase production. Addition of 1% (w/v) lactose or glucose (both from Sigma Aldrich) was used as additional controls. Escherichia coli JM109 was used for cloning (Yanisch‐Perron *et al*., 1985).

#### Nucleic acid isolation and transcript analysis

Isolation of total RNA was performed using the RNAeasy plant mini kit (QIAGEN, Hilden, Germany) as described previously (Tisch *et al*., 2011a). Estimation of crossing partner contamination was evaluated by the relative abundance of mating type genes in co‐precipitated chromosomal DNA in the RNA samples (Seibel *et al*., 2012b). Contamination was generally lower than 1.02% and did not influence the results presented. The integrity of extracted RNA was first evaluated by agarose gel electrophoresis and staining with SYBR^®^ Safe DNA (Invitrogen, Carlsbad, USA). The concentration and purity of extracted RNA were analyzed using the Nanodrop ND‐1000 spectrophotometer (PEQLAB, Erlangen, Germany). The quality of samples was further confirmed on the Agilent 2100 Bioanalyzer platform using the RNA 6000 Nano Kit (Agilent, Santa Clara, USA) according to the manufacturer's instructions. Only samples with a RIN (RNA integrity number) > 9 were taken for cDNA preparation (Fleige and Pfaffl, 2006). One microgram of total RNA was treated with DNAaseI (Thermo Fisher/Fermentas, St. Leon‐Rot, Germany) for 30 min. The DNAaseI treatment was terminated by adding EDTA to a final concentration of 2.5 mM and incubation at 65°C for 10 min. The RevertAid First Strand cDNA Synthesis Kit (Thermo Fisher/Fermentas) was used for first‐strand cDNA synthesis according to the manufacturer's protocol, and cDNA was diluted prior to qRT‐PCR. No‐template controls and RT‐minus controls (reverse transcription omitted) were included. qPCR reactions were performed in a CFX96 Real‐Time PCR Detection System (Bio‐Rad Laboratories GmbH, Hercules, USA) using the iQ SYBR Green Supermix (Bio‐Rad Laboratories GmbH). Gene‐specific primers used in this study are summarized in Supporting Information Table S1. *rpl6e* encoding the ribosomal protein RPL6e was used as reference gene. Transcription of *rpl6e* was demonstrated to be unaffected by light or light regulators (Tisch *et al*., 2011a). Melting curves after the qPCR were evaluated to confirm that the signal was the result of single‐product amplification and not due to primer dimers or arbitrary amplification. Cycle threshold values were determined for a minimum of two biological replicates and three technical replicates. All data were evaluated and analyzed with the qbase+ software package (Biogazelle, Gent, Belgium) (Hellemans *et al*., 2007). Statistical analysis of the qPCR data was performed with the qbase + (analysis of variance, Tukey–Kramer post‐test, *P* < 0.05).

#### Construction of deletion strains

In order to delete *env1*, plasmid pDELENV2 (Castellanos *et al*., 2010) was used. The deletion cassette was released from pDELENV2, using restriction enzymes *Acc*65I and *Bam*HI (Thermo Fisher/Fermentas), and was used to transform protoplasts (Gruber *et al*., 1990). Selection of colonies was performed on 3% (w/v) malt extract agar plates containing 100 μg ml^−1^ hygromycin B (Roth). The deletion strains were checked using PCR primers binding outside the deleted region (ENVScreen_F1 and ENVScreenR1) resulting in considerably longer fragments in mutant strains compared with the wild type.

To construct pDELVEL1 for deletion of *vel1*, a 1644 bp fragment spanning 5′ region of the gene was amplified using primers vel5F and vel5R. Similarly, a 922 bp fragment flanking the 3′ region of the gene was amplified using primers vel3F and vel3R. The marker construct containing the *amdS* gene (Penttila *et al*., 1987) was amplified using primers amdSF and amdSR. The prepared fragments were transformed into yeast and the construction of the vector performed by yeast recombination (Colot *et al*., 2006). *T. reesei* transformation was done as described above with the deletion cassette amplified from the vector using vel5F and vel3R. Successful homologous integration in transformants was analyzed using primers ScreenVel_AF1 and ScreenVel_AR1. The copy number of the deletion cassettes in the deletion strains was analyzed using qPCR as described previously (Tisch *et al*., 2011b). Strains comprising one deletion cassette on the homologous locus were used for further analysis.

#### Microscopy analysis

Agar block microscopy was performed as described previously (Woo *et al*., 2010). Briefly, strains were grown on 3% (w/v) malt extract agar at 22°C. Agar blocks were cut using a dissecting knife from the edge of the colony and were subsequently placed on a glass slide. A drop of lactophenol blue stain (Sigma) and a coverslip were put onto the agar block. Samples were immediately examined under a Nikon Eclipse E200 light microscope (Nikon, Tokyo, Japan).

#### Analysis of secondary metabolites

Thin‐layer chromatography (TLC) analysis was performed as described earlier (Bok and Keller, 2004; Reyes‐Dominguez *et al*., 2010) with several modifications: Briefly, samples were collected from agar plates in the same size, growth stage and location as used for qRT‐PCR (Fig. 3). Agar pieces from at least three plates were pooled and two independent biological replicates were made. Samples were homogenized in liquid nitrogen and resuspended in acetone : water (1:1; v/v; chemicals from Roth). For extraction of metabolites from mycelia and agar, samples were stored at 4°C for several hours and thereafter chloroform was added. The organic phase (chloroform) was transferred to a conical tube. After complete evaporation of the solvent, the samples were resuspended in 160 μl chloroform and 8 μl was spotted on a TLC plate (HPTLC silica gel 60 F254s, Merck 1.15696.0001) using the CAMAG Automatic TLC sampler 4 (CAMAG, Muttenz, Switzerland). This amount was chosen in order to detect smaller changes in metabolite patterns without overloading the plate. Separation was performed in a saturated twin trough chamber with chloroform : formic acid 7:1 (v/v). The plates were analyzed under ultraviolet light (254 nm and 366 nm) using a CAMAG visualizer (CAMAG). Additionally, the plates were derivatized with p‐anisaldehyde : sulfuric acid and evaluated again with white and ultraviolet light. Results were visualized using the software visionCATS 1.4.14017.1 (CAMAG).

#### Bioinformatic analyses

Blast analyses were done using the NCBI Blast homepage (http://blast.st‐va.ncbi.nlm.nih.gov/Blast.cgi), AspGD (http://www.aspgd.org/) and JGI MycoCosm (http://genome.jgi‐psf.org/programs/fungi/index.jsf). PEST motifs (Rechsteiner and Rogers, 1996) were determined using ePESTfind (http://emboss.bioinformatics.nl/cgi‐bin/emboss/epestfind) and nuclear export sequences were searched using the NetNES1.1 server (la Cour *et al*., 2004); http://www.cbs.dtu.dk/services/NetNES/). PsortII (http://psort.hgc.jp/form2.html) was used to identify nuclear localization sequences.

For phylogenetic analysis, the alignment was done with clustalX, and minimum evolution analysis was performed using MEGA4 (2007) with 500 bootstrap cycles.

## Supporting information

Supporting information
