# Supplementary material for: Mating type‐dependent partner sensing as mediated by VEL1 in T richoderma reesei
Source: Mol Microbiol. 2015 Apr 16;96(6):1103–18. doi: 10.1111/mmi.12993 (PMC4949666; doi:10.1111/mmi.12993)
Supplement: Supplementary file 1 — Supporting information [file MMI-96-1103-s001.pdf]

Mating type dependent partner sensing as mediated by VEL1 in *Trichoderma reesei*

Hoda Bazafkan[1], Christoph Dattenböck[1], Stefan Böhmdorfer[2], Doris Tisch[3], Eva Stappler[1] and Monika Schmoll[1]*

Supporting information

APPENDIX S1 - The velvet family proteins in *Trichoderma* spp

We analyzed the publicly available genomes of 6 *Trichoderma* species for the presence of members of the velvet family of proteins (Bayram & Braus, 2012). Bidirectional Blast analyses and phylogenetic analysis of members of this group in several fungi including *Aspergillus* spp., *Fusarium* spp., *Botryotinia fuckeliana* and *Neurospora crassa* revealed that *Trichoderma* spp. contain orthologues of *A. nidulans* VeA, VelB and VelC, but no VosA (supplementary figure S1). The *T. reesei* orthologues are represented by TR_122284 (VEL1/VeA; e-value 9e-48, 18 % sequence identity), TR_40551 (VEL2/VelB) and TR_102737 (VEL3/VelC). Conserved Domain Search at the NCBI server showed the presence of the velvet domain (Pfam11754), with e-values of 1.17e-87 for VEL1, 2.95e-46 for VEL2 and 2.34e-49 for VEL3. Interestingly, the velvet domain in VEL2 is not split as found in *A. nidulans* VelB (Chang *et al.*, 2013). The nuclear localization signal (NLS) of *A. nidulans* (Kim *et al.*, 2002) is conserved in *Trichoderma* spp. but only a weak nuclear export sequence (NES) was detected, albeit more downstream than in *A. nidulans*, i. e. around amino acid 448 in *T. reesei*. Analysis for PEST sequences, reflecting instability of a protein and representing potential proteolytic cleavage sites (Rechsteiner & Rogers, 1996) revealed that *Trichoderma* spp. do contain such sequences, but their position is not conserved between *A. nidulans* and *Trichoderma*. In *T. reesei*, this sequence is found between positions 290 and 311 (HAPPPLPPPPPSSYDAPPPAAR).

Supplementary Figure S1. Phylogenetic analysis of VELVET family proteins in *Trichoderma* spp.

The phylogenetic tree was obtained using MEGA4.0 with the minimum evolution method. Numbers at branches indicate their bootstrap support values. Locus IDs or protein IDs are given along with abbreviated species names representing *Trichoderma reesei*, *Trichoderma atroviride, Trichoderma virens, Trichoderma longibrachiatum, Trichoderma asperellum, Trichoderma heazianum, Fusarium graminearum, Fusarium verticillioides, Fusarium fujikuroi, Botryotinia fuckeliana, Neurospora crassa, Magnaporthe oryzae, Aspergillus nidulans, Aspergillus niger, Aspergillus fumigatus, Aspergillus flavus, Aspergillus clavatus* and *Aspergillus sydowii*. Arrows indicate the *T. reesei* homologue of the respective groups.

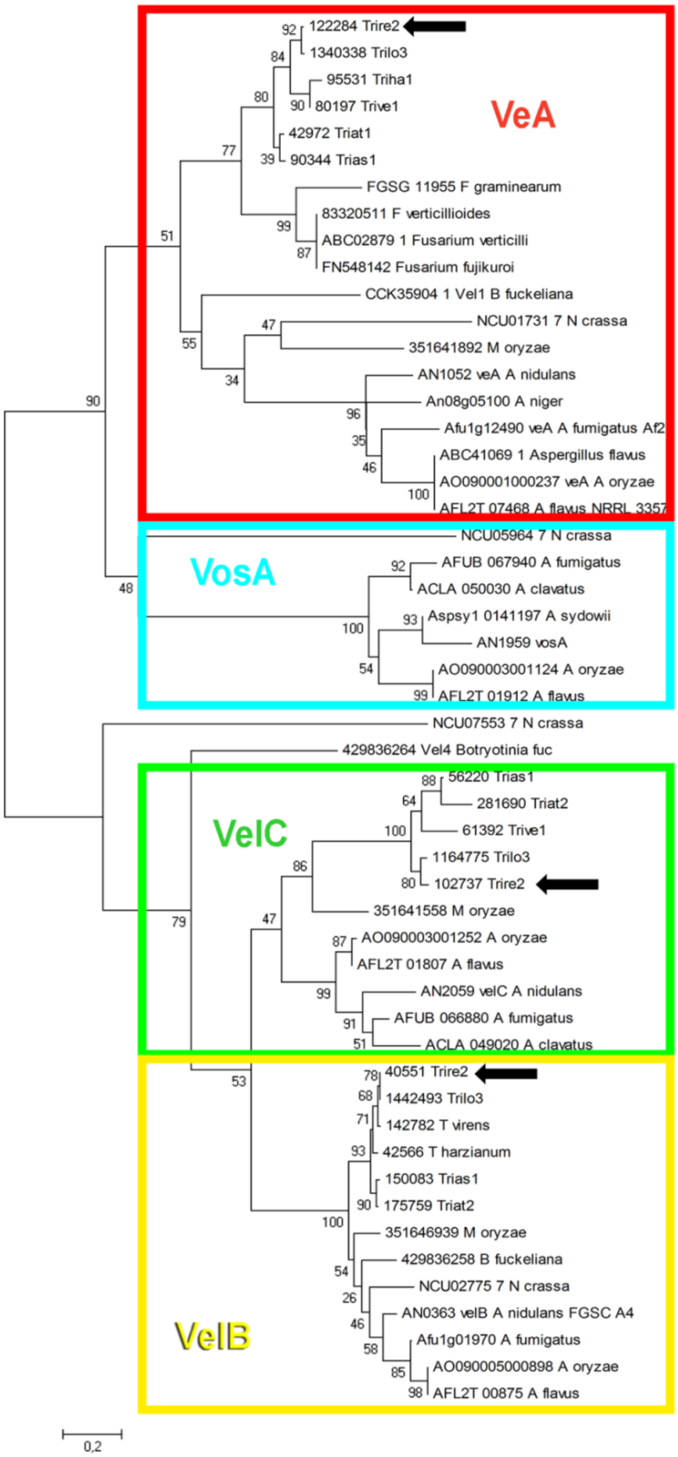

APPENDIX S2 - VEL1 impacts growth, conidiation and cellulase expression in a female fertile background

We analyzed whether alternative carbon sources in combination with different light and temperature conditions would induce conidiation. We applied D-galactose, D-sorbitol, D-mannitol, D-arabinose, meso-erythritol and γ-amino butyric acid (GABA) as these carbon sources are known to induce strong conidiation in *Trichoderma atroviride* (Friedl *et al.*, 2008). Growth and conidiation on these carbon sources were investigated in constant light, constant darkness, daylight (12:12 cycles), constant red light, with heat shocks (50 °C) for 2, 4, or 6 hours or cold shock (4°C) over night. Shock conditions were applied after three days of growth under the light conditions specified. Although QM6a sporulated readily under these conditions (with the exception of meso-erythritol), strains lacking *vel1* did not. Xylose is also known to be beneficial for conidiation, and mechanical injury was shown to induce conidiation in *T. atroviride* (Hernandez-Onate *et al.*, 2012). Still, conidiation of Δ*vel1* was not achieved on xylose either. Hence we conclude that VEL1 is essential for conidiation in *T. reesei*.

Agar-block microscopy from strains grown on 3% (w/v) malt extract for 48 h under daylight and constant darkness was performed. Deletion of *vel1* neither influenced hyphal diameter nor hyphal branching compared to the wildtype (data not shown).

Growth on plates was reduced in Δ*vel1* on carboxymethylcellulose (CMC) in constant light (to 46 ± 10 %) and darkness (to 36 ± 5 %) compared to wild-type. Hence we also applied the Congo-Red screening test (Carder, 1986) for production of cellulases on plates and found that degradation of CMC was not detectable for Δ*vel1*. Addition of lactose to overcome growth limitation on cellulose due to lack of cellulase production also did not alleviate the growth defect (darkness: 54 ± 5 % and light: 37 ± 3 %). Again no degradation of CMC was observed although lactose induces cellulase production in the wild-type. Interestingly, addition of glucose to the CMC medium, while meant to serve as control, did rescue the growth defect of Δ*vel1* specifically in light and when grown in 12:12 light-dark cycles (83 ± 6 %). Consequently, we assume that the growth defect of Δ*vel1* could be due to altered adaptation of carbon sensing to different light conditions. Residual growth on cellulose is likely to be due to utilization of other nutrients in the medium (for example components of the phosphate-citrate buffer) or limited degradation of CMC that did not significantly affect Congo-Red staining.

Supplementary Figure S2. Secondary metabolite analysis, original pictures.
The different panels show original pictures with different visualization techniques, which allow for detection of different substance classes in the sample. (A) Remission at 366 nm, (B) Derivatized with anisaldehyde – sulfuric acid, transmission, visual light, (C) Remission at 254 nm, (D) Derivatized with anisaldehyde – sulfuric acid, remission at 254 nm, (E) Remission, transmission at visual light. Sample codes are summarized in Figure 3, uninoculated medium (M) was used as control.

A

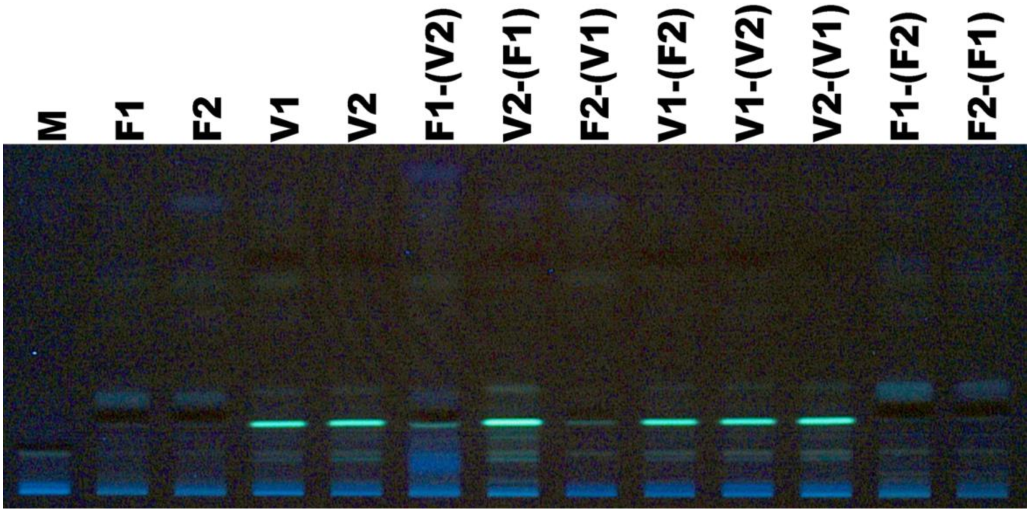

B

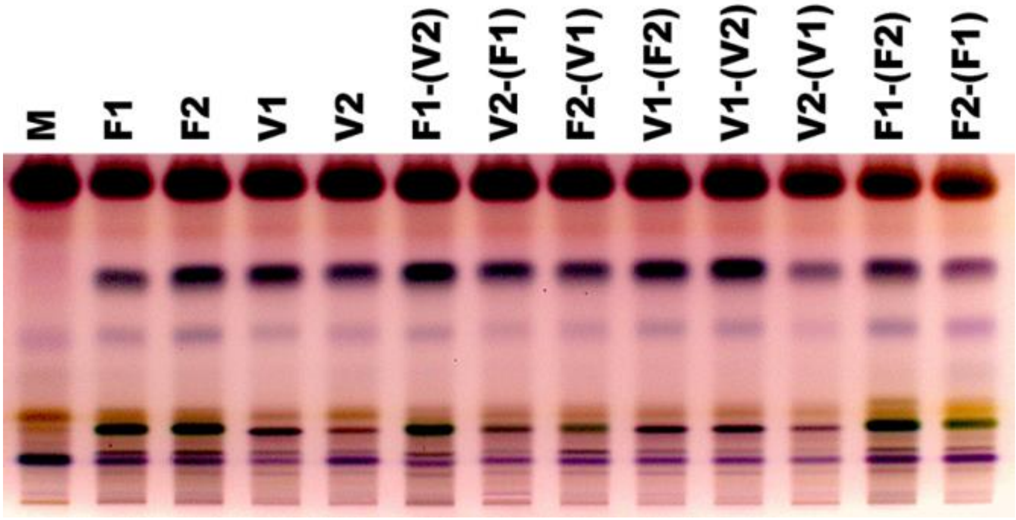

C

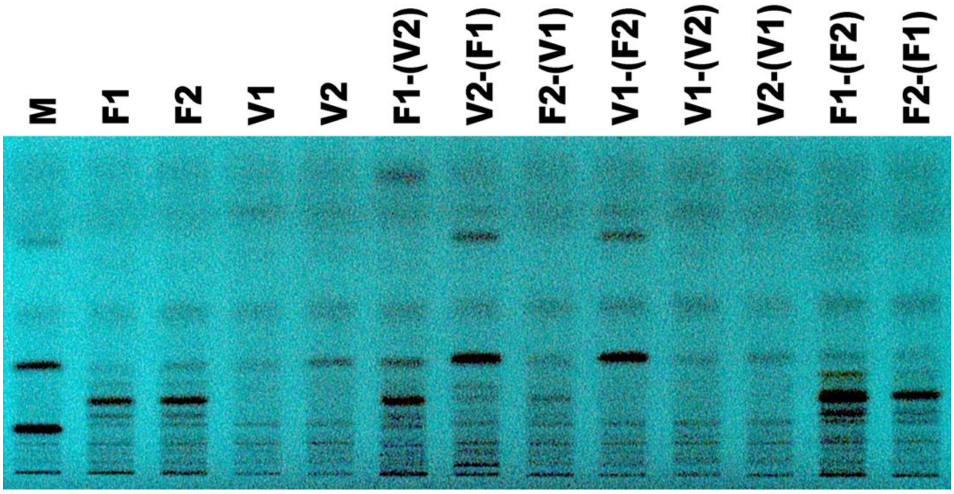
M
F1
F2
V1
V2
F1-(V2)
V2-(F1)
F2-(V1)
V1-(F2)
V1-(V2)
V2-(V1)
F1-(F2)
F2-(F1)

D

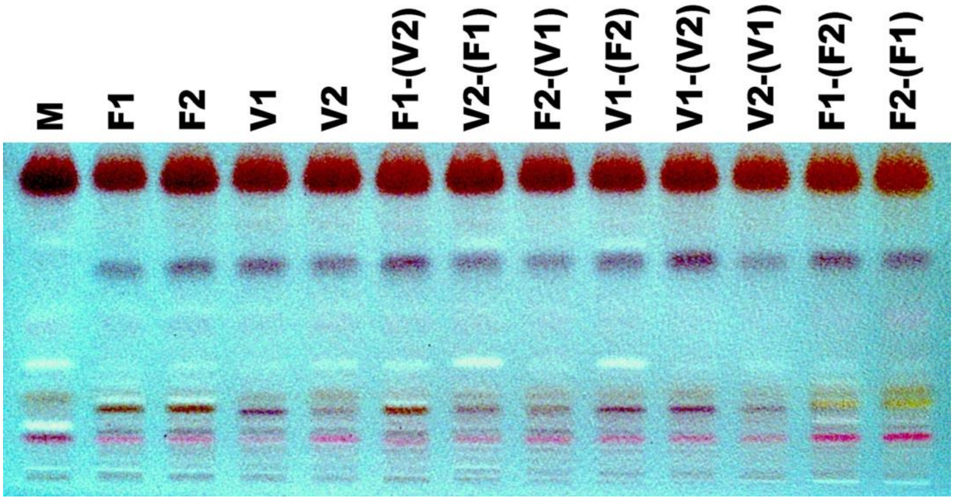
M
F1
F2
V1
V2
F1-(V2)
V2-(F1)
F2-(V1)
V1-(F2)
V1-(V2)
V2-(V1)
F1-(F2)
F2-(F1)

E

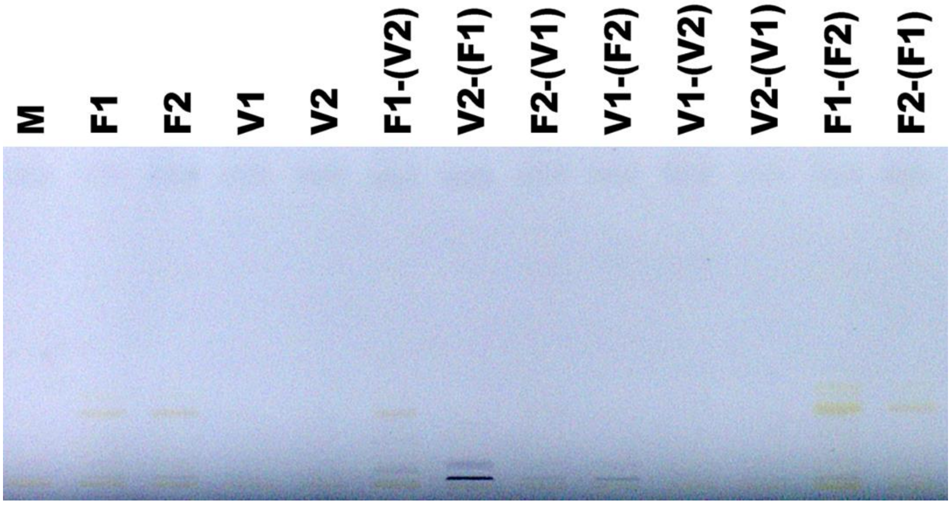
M
F1
F2
V1
V2
F1-(V2)
V2-(F1)
F2-(V1)
V1-(F2)
V1-(V2)
V2-(V1)
F1-(F2)
F2-(F1)

Supplementary table S1 Oligonucleotides used in this study

Oligo-nucleotide	Sequence	Purpose	Reference
vel5F	5′ GTAACGCCAGGGTTTTCCCAGT CACGACGAGAGAGAGAGCAGGA AAAGG 3′	*vel1* deletion vector	This study
vel5R	5′ GATGACATTCATACTCAAGACG TCGACCTCGAGAAGCCAAACAGA CATC 3′	*vel1* deletion vector	This study
vel3F	5′ GGAGCTCCAGCTTTTGTTCCCTT TAGTG AGGACCAGTACCCTTGT TC 3′	*vel1* deletion vector	This study
vel3R	5′ GCGGATAACAATTTCACACAGGA AACAGCTCCCTTCTTCCTCTTCAC 3′	*vel1* deletion vector	This study
ScreenVel_AF1	5′ GGGATTTGCGGCTAACAC 3′	Screening deletion of *vel1*	This study
ScreenVel_AR1	5′ CATTGCGAAGAGAAACCAGG 3′	Screening deletion of *vel1*	This study
ENVScreen_F1	5′ ACTGCGTGTTACCAAATG 3′	Screening deletion of *env1*	This study
ENVScreen_R1	5′ CAACATGAACCTGGAAGC 3′	Screening deletion of *env1*	This study
RT_amdS_F1	5′ CACCAGGGCTACGAAACATC 3′	Copy number of *vel1* deletion construct	This study
RT_amdS_R1	5′ GCCCGATGATGTTGTTGAC 3′	Copy number of *vel1* deletion construct	This study
ppg1_c_qF	5′ CCGTCCTGAGCGCCACCATT 3′	qRT-PCR *ppg1*	This study
ppg1_c_qR	5′ CGATGACGCTGCGAGCAACG 3′	qRT-PCR *ppg1*	This study
hpr1_c_qF	5′ ATCCGCTTCCGCCAAGTCAC 3′	qRT-PCR *hpr1*	This study
hpr1_c_qR	5′ CAGGGGGTGGACGAGGATGA 3′	qRT-PCR *hpr1*	This study
RT_VEL_F1	5′ CGAGGAGGGCAAGGACATTAC 3′	qRT-PCR *vel1*	This study
RT_VEL_R1	5′ GCAGGAACACCAGTCAGGATG 3′	qRT-PCR *vel1*	This study
amdSF	5′ AGGTCGACGTCTTGAGTATG 3′	marker construct	This study
amdSR	5′ TCACTAAAGGGAACAAAAGC 3′	marker construct	This study
hpp1F	5′ ACAATCACCGTGGGACATCTG 3′	qRT-PCR *hpp1*	(Seibel *et al.*, 2012b)
hpp1R	5′ TCCCTGCTGTTCCGCTGATG 3′	qRT-PCR *hpp1*	(Seibel *et al.*, 2012b)
hpr2F	5′ TGGCACCACTTCATCAACTTC 3′	qRT-PCR *hpr2*	(Seibel *et al.*, 2012b)
hpr2R	5′ GGAGTAGGAGGAGGATGTGTTG 3′	qRT-PCR *hpr2*	(Seibel *et al.*, 2012b)

REFERENCES

Bayram, O. & G.H. Braus, (2012) Coordination of secondary metabolism and development in fungi: the velvet family of regulatory proteins. *FEMS Microbiol Rev* **36**: 1-24.

Carder, J.H., (1986) Detection and quantitation of cellulase by Congo red staining of substrates in a cup-plate diffusion assay. *Anal Biochem* **153**: 75-79.

Chang, P.K., L.L. Scharfenstein, P. Li & K.C. Ehrlich, (2013) *Aspergillus flavus* VelB acts distinctly from VeA in conidiation and may coordinate with FluG to modulate sclerotial production. *Fungal Genet Biol* **58-59**: 71-79.

Friedl, M.A., M. Schmoll, C.P. Kubicek & I.S. Druzhinina, (2008) Photostimulation of *Hypocrea atroviridis* growth occurs due to a cross-talk of carbon metabolism, blue light receptors and response to oxidative stress. *Microbiology* **154**: 1229-1241.

Hernandez-Onate, M.A., E.U. Esquivel-Naranjo, A. Mendoza-Mendoza, A. Stewart & A.H. Herrera-Estrella, (2012) An injury-response mechanism conserved across kingdoms determines entry of the fungus *Trichoderma atroviride* into development. *Proc Natl Acad Sci U S A* **109**: 14918-14923.

Kim, H., K. Han, K. Kim, D. Han, K. Jahng & K. Chae, (2002) The *veA* gene activates sexual development in *Aspergillus nidulans*. *Fungal Genet Biol* **37**: 72-80.

Rechsteiner, M. & S.W. Rogers, (1996) PEST sequences and regulation by proteolysis. *Trends Biochem Sci* **21**: 267-271.

Schmoll, M., L. Franchi & C.P. Kubicek, (2005) Envoy, a PAS/LOV domain protein of *Hypocrea jecorina* (Anamorph *Trichoderma reesei*), modulates cellulase gene transcription in response to light. *Eukaryot Cell* **4**: 1998-2007.

Seibel, C., D. Tisch, C.P. Kubicek & M. Schmoll, (2012a) ENVOY is a major determinant in regulation of sexual development in *Hypocrea jecorina* (*Trichoderma reesei*). *Eukaryot Cell* **11**: 885-890.

Seibel, C., D. Tisch, C.P. Kubicek & M. Schmoll, (2012b) The role of pheromone receptors for communication and mating in *Hypocrea jecorina (Trichoderma reesei)*. *Fungal Genet Biol* **49**: 814-824.

Tisch, D., C.P. Kubicek & M. Schmoll, (2011) The phosducin-like protein PhLP1 impacts regulation of glycoside hydrolases and light response in *Trichoderma reesei*. *BMC Genomics* **12**: 613.
